# Temporal changes and reference values for adolescent handgrip strength in the Republic of Korea: A 9-year analysis of the KNHANES cohort

**DOI:** 10.1371/journal.pone.0357474

**Published:** 2026-09-03

**Authors:** Jozo Grgic, Vanessa Kristina Wazny, Dorian Varovic

**Affiliations:** 1 Department of Sports Science and Physical Education, Faculty of Education, The Chinese University of Hong Kong, Hong Kong, China; 2 NUS Academy for Healthy Longevity, Yong Loo Lin School of Medicine, National University of Singapore (NUS), Singapore, Singapore; 3 Department of General and Applied Kinesiology, Faculty of Kinesiology, University of Zagreb, Zagreb, Croatia; Mongolian National University of Medical Sciences, MONGOLIA

## Abstract

We aimed to examine temporal changes in handgrip strength and develop reference values among adolescents (10–19 years; *n* = 4,867) in the Republic of Korea. A general linear model was used to explore differences in absolute and body size normalized (height and body mass) handgrip strength across survey years using data from KNHANES 2014–2022. Weighted percentiles were calculated to generate reference values. In some of the male age groups, handgrip strength was lower in 2016–2019 (−1.7 to −4.1 kg; −0.197 to −0.267 units) and higher in 2014–2015 (2.6 to 3.5 kg; 0.175 to 0.309 units), compared to 2022. In some of the female age groups, handgrip strength was lower in 2016–2019 (−1.6 to −2.6 kg; −0.123 to −0.302 units) and higher in 2014–2015 (1.4 kg; 0.178 to 0.316 units), compared to 2022. Reference values were lowest among 10–11‑year‑olds, showed progressive increases across older age groups, and differed according to sex. Overall, handgrip strength remained fairly stable over the 9-year period, even though in some of the years it was lower or higher compared to the most recent wave. However, these differences were generally small, did not occur at every survey year, and were completely absent for some of the sex and age groups. A similar pattern was observed for normalized values, although the results were not entirely consistent with absolute handgrip strength data. The provided reference values enable interpretation of absolute and body size normalized handgrip strength in Korean adolescents.

## Introduction

Handgrip strength is a well-established biomarker of overall health [1]. It has been extensively studied in older adults due to its strong associations with mortality, frailty, and sarcopenia [1–4]. However, the relevance of handgrip strength extends to younger populations as well [5–9]. Among adolescents, handgrip strength has been associated with a wide range of outcomes, including cardiovascular health, bone mineral content, overall muscle strength, nutritional status, academic performance, and mental health [5–9]. Handgrip strength is also included in the Youth Fitness International Test and the EUROFIT test battery [10,11].

Recent research has examined temporal changes in fitness-related outcomes as these patterns provide insight into broader changes in population health [12,13]. Monitoring such changes is also valuable for public health surveillance, developing preventive strategies, and evaluating policies [12,13]. A large-scale analysis of adolescent handgrip strength from 1967 to 2017 reported overall improvements over time [13]. However, these findings may not accurately reflect current trends, as more recent data indicate declines in physical activity participation, with approximately 80% of adolescents failing to meet activity guidelines [14]. Thus, historical trends may not be generalizable to today’s adolescent populations.

To interpret handgrip strength at the individual level, clinicians and researchers require reference (normative) values that enable comparisons with age- and sex-specific populations [15]. Most existing studies have focused on Western populations, limiting their applicability to other regions, such as Asia [16–18]. Specifically, as recently shown for adults, reference values for Europeans are generally higher compared to their Asian counterparts [19,20]. Reference values are most robust when derived from stable, multi‑year frameworks like national cohort surveys. However, because research reports variation in handgrip strength over time [13], reference values based on multiple survey waves should ideally include an analysis of year‑to‑year changes.

Korea National Health and Nutrition Examination Survey (KNHANES) is an ongoing, nationally representative cohort designed to assess the health status, health behaviours, and nutritional intake of the population in the Republic of Korea [21]. The KNHANES provides a unique opportunity to explore temporal changes and establish reference values given the use of standardized data collection protocols as well as its national representativeness. Accordingly, using the KNHANES data, the objectives of this study were to: (a) examine temporal changes in handgrip strength among adolescents; and (b) develop handgrip strength reference values for the adolescent population in the Republic of Korea.

## Materials and methods

### Study design and participants

The KNHANES employs a stratified, multi-stage, cluster-based probability sampling method to obtain a representative sample of South Korea’s non-institutionalized civilian population [21]. Ethical approval for the cohort was granted by the Research Ethics Review Committee of the Korea Disease Control and Prevention Agency [21]. Although the survey began in 1998, handgrip strength measurement was incorporated in 2014 and has been consistently assessed in subsequent waves [22]. Handgrip strength was assessed in waves 2014–2019. After 2019, handgrip strength was again evaluated in 2022 [22]. The total pooled sample size for this study was 4,867 participants aged 10–19 years. To allow for granularity in the data, the sample was categorized into two-year age groups, starting with 10–11 years (male *n* = 619; female *n* = 510), 12–13 years (male *n* = 558; female *n* = 473), 14–15 years (male *n* = 502; female *n* = 457), 16–17 years (male *n* = 458; female *n* = 448), and 18–19 years (male *n* = 437; female *n* = 405). The participants with missing handgrip data were not included in this study.

### Handgrip strength

Handgrip strength was assessed using a digital hand dynamometer (Takei, Tokyo, Japan). This device can measure forces of up to 100 kg in 0.1 kg increments and features an adjustable grip span [23,24]. For the test, participants were required to stand upright with their feet hip-width apart, look forward, and keep their elbows fully extended. Upon receiving commands from the assessors, the participants were required to squeeze the dynamometer with maximal effort. The testing started with the dominant hand. Testing was performed three times on both hands, with a 60 second rest interval between trials. In waves 2014–2019, the testing was performed with six allowed attempts, which was reduced to four (two attempts each hand) in wave 2022. To account for this methodological difference, in the primary analyses we considered only the first 4 attempts (two attempts each hand) from waves 2014–2019. However, to examine the consistency of the findings, secondary analyses were performed with all six attempts. In all waves, the maximum value across trials was used as the handgrip strength data, in line with the current recommendations [25]. Besides absolute handgrip strength, we also normalized handgrip strength for body size, using the methods proposed by Nevill et al. [26]. Specifically, normalized handgrip strength was calculated as: handgrip strength in kilograms/(height in meters^2^ * body mass in kilograms^0.333^) and is referred to as “units” in the following sections for brevity.

### Statistical analysis

All analyses were conducted separately for males and females across the following age groups: 10–11 years, 12–13 years, 14–15 years, 16–17 years, and 18–19 years. To account for the complex survey design, including stratification, clustering, and unequal probabilities of selection, analyses incorporated the sample weights, strata, and primary sampling units (PSUs) specified in the dataset. To account for the independent cross‑sectional design of KNHANES waves (2014–2022), unique identifiers were created to combine survey year with the original strata and PSU variables. Differences in absolute and normalized handgrip strength were examined using the Complex Samples General Linear Model (CSGLM). Handgrip strength was the dependent variable while survey year was a categorical independent variable. Normality of distribution of the residuals was explored using histograms and Q-Q plots. The residuals were either normally distributed or had a slight negative skew, but we proceeded with the analyses in the latter case given that CSGLM is robust to mild non-normality. In all analyses, there was no substantial heteroscedasticity. When a significant omnibus effect was found, pairwise comparisons were performed for the estimated marginal means. All comparisons were made relative to the most recent survey wave (2022). Secondary analyses were performed while considering all six attempts in survey years 2014–2019. To establish reference values, weighted percentiles (5th, 10th, 20th, 30th, 40th, 50th, 60th, 70th, 80th, 90th, 95th percentile) were calculated. In the primary analysis, reference values were derived from data pooled across all available survey waves (2014–2019 and 2022). In the secondary analysis, reference values were calculated using only the most recent survey wave (2022). The statistical significance threshold was set at *p* < 0.05. Statistical analyses were performed using SPSS v. 30 (IBM, Chicago, IL, USA).

## Results

### Male handgrip strength

Significant omnibus effects across survey years were found for males of all age groups (*p* = 0.019 to < 0.001), except 18–19 years (*p* = 0.498).

Handgrip strength was lower in: (a) 2019 for 10–11-year-olds and 12–13-year-olds (−2.2 and −2.9 kg; *p* = 0.005 and 0.001); (b) 2018 for 10–11-year-olds, 12–13-year-olds, and 16–17-year-olds (−1.7 to −4.1 kg; *p* = 0.019 to 0.004); and (c) 2016 for 16–17-year-olds (−4.1 kg; *p* = 0.013; Table 1).

**Table 1:** Male participant characteristics according to survey year (*n* = 2,574).

Year	*n*	Height (cm)	Weight (kg)	Handgrip strength (kg)	Normalized handgrip strength (kg/(m^2^ * kg^0.333^)
10–11 years					
2014	88	146.1 ± 6.4	40.6 ± 8.3	17.5 (16.7, 18.3)	2.394 (2.299, 2.498)**
2015	91	147.3 ± 7.9	44.3 ± 11.9	18.5 (17.6, 19.3)	2.405 (2.330, 2.480)**
2016	89	145.9 ± 7.2	41.8 ± 10.9	16.7 (15.8, 17.6)	2.261 (2.200, 2.322)
2017	85	148.9 ± 7.7	43.9 ± 10.6	17.2 (16.3, 18.0)	2.195 (2.105, 2.285)
2018	98	146.5 ± 8.2	44.0 ± 11.3	15.9 (15.0, 16.8)*	2.118 (2.023, 2.214)
2019	100	146.6 ± 7.2	42.2 ± 8.5	15.5 (14.8, 16.2)*	2.070 (1.990, 2.150)
2022	68	149.5 ± 7.2	48.0 ± 12.0	17.6 (16.9, 18.4)	2.183 (2.101, 2.265)
12–13 years					
2014	71	161.9 ± 7.8	53.9 ± 12.8	27.4 (25.6, 28.9)	2.760 (2.632, 2.887)
2015	87	160.4 ± 9.3	55.2 ± 13.2	26.0 (24.3, 27.6)	2.622 (2.512, 2.732)
2016	91	163.1 ± 7.9	54.1 ± 12.7	25.8 (24.3, 27.2)	2.561 (2.423, 2.698)
2017	93	161.9 ± 7.9	52.3 ± 10.8	25.0 (23.6, 26.4)	2.521 (2.426, 2.616)
2018	82	162.3 ± 8.6	54.2 ± 11.7	23.8 (21.8, 25.8)*	2.367 (2.240, 2.495)*
2019	80	161.3 ± 9.2	58.2 ± 14.1	24.0 (22.6, 25.5)*	2.379 (2.266, 2.491)*
2022	54	163.1 ± 7.5	58.8 ± 14.7	27.0 (25.5, 28.4)	2.634 (2.492, 2.776)
14–15 years					
2014	89	170.5 ± 6.1	65.0 ± 14.2	36.4 (35.0, 37.8)**	3.131 (3.032, 3.239)**
2015	86	169.1 ± 6.3	61.9 ± 12.3	35.4 (33.9, 36.9)**	3.137 (3.034, 3.240)**
2016	76	171.0 ± 7.0	65.1 ± 13.3	32.9 (31.4, 34.5)	2.811 (2.666, 2.956)
2017	63	171.4 ± 7.2	65.2 ± 13.6	33.4 (31.7, 35.2)	2.833 (2.721, 2.945)
2018	64	172.9 ± 5.7	65.9 ± 14.0	31.9 (30.7, 33.1)	2.681 (2.566, 2.796)
2019	71	171.1 ± 5.9	65.7 ± 15.1	33.4 (32.0, 34.8)	2.851 (2.746, 2.956)
2022	53	170.7 ± 6.2	63.6 ± 12.7	32.8 (31.2, 34.5)	2.828 (2.706, 2.950)
16–17 years					
2014	68	172.3 ± 6.5	66.6 ± 12.5	39.9 (38.4, 41.4)	3.336 (3.239, 3.433)**
2015	66	174.0 ± 6.1	69.0 ± 16.4	40.0 (38.1, 41.9)	3.259 (3.091, 3.426)
2016	74	172.9 ± 6.1	64.2 ± 12.4	35.3 (33.7, 36.8)*	2.968 (2.827, 3.110)
2017	80	173.4 ± 5.5	66.5 ± 11.2	38.1 (36.5, 39.6)	3.144 (3.017, 3.270)
2018	68	173.2 ± 5.8	70.4 ± 15.6	35.3 (33.5, 37.2)*	2.868 (2.758, 2.978)*
2019	61	175.9 ± 6.2	68.9 ± 12.7	36.8 (35.4, 38.2)	2.930 (2.817, 3.044)*
2022	41	175.4 ± 6.8	70.2 ± 12.5	39.4 (36.5, 42.3)	3.127 (2.993, 3.262)
18–19 years					
2014	62	173.1 ± 5.9	68.9 ± 13.8	39.2 (38.2, 40.2)	3.223 (3.132, 3.314)**
2015	83	173.6 ± 5.1	68.6 ± 13.1	40.2 (38.5, 42.0)	3.276 (3.137, 3.415)**
2016	65	173.1 ± 6.2	72.1 ± 16.0	37.9 (36.3, 39.5)	3.060 (2.952, 3.168)
2017	60	175.3 ± 6.3	68.1 ± 11.7	39.6 (37.7, 41.5)	3.162 (3.064, 3.261)
2018	62	176.0 ± 4.7	69.6 ± 12.7	38.0 (36.5, 39.6)	3.004 (2.882, 3.126)
2019	67	175.3 ± 5.1	72.7 ± 15.8	38.5 (36.5, 40.6)	3.023 (2.877, 3.169)
2022	38	175.3 ± 7.1	72.4 ± 14.8	38.9 (36.6, 41.1)	3.048 (2.938, 3.159)

Data are presented as mean ± standard deviation or marginal mean (95% confidence interval); * denotes statistically lower values compared to 2022 (reference year); ** denotes statistically higher values compared to 2022 (reference year).

Handgrip strength was higher in 2015 and 2014 (2.6 and 3.5 kg; *p* = 0.022 and 0.001) for 14–15-year-olds.

### Female handgrip strength

Significant omnibus effects across survey years were found for females of all age groups (*p* = 0.003 to < 0.001), except 14–15 years (*p* = 0.061).

Handgrip strength was lower in: (a) 2019 for 10–11-year-olds, 16–17-year-olds, and 18–19-year-olds (−1.6 to −1.9 kg; *p* = 0.015 to 0.002); (b) 2018 for 10–11-year-olds, 12–13-year-olds, 16–17-year-olds, and 18–19-year-olds (−2.3 to −2.6 kg; *p* = 0.020 to < 0.001); (c) 2017 for 10–11-year-olds and 12–13-year-olds (−1.3 to −1.7 kg; *p* = 0.036 to 0.017); and (d) 2016 for 10–11-year-olds (−1.7 kg; *p* = 0.012; Table 2).

**Table 2:** Female participant characteristics according to survey year (*n* = 2,293).

Year	*n*	Height (cm)	Weight (kg)	Handgrip strength (kg)	Normalized handgrip strength (kg/(m^2^ * kg^0.333^)
10–11 years					
2014	69	146.5 ± 7.4	40.1 ± 9.9	16.5 (15.3, 17.6)	2.224 (2.125, 2.323)
2015	60	147.2 ± 7.1	41.4 ± 11.0	17.1 (15.8, 18.3)	2.274 (2.152, 2.395)
2016	78	146.9 ± 7.2	39.7 ± 9.1	15.3 (14.5, 16.0)*	2.079 (1.991, 2.167)*
2017	91	146.8 ± 7.3	40.1 ± 9.8	15.6 (14.9, 16.3)*	2.115 (2.042, 2.189)
2018	75	148.9 ± 7.1	40.3 ± 7.5	14.6 (13.5, 15.7)*	1.909 (1.793, 2.025)*
2019	76	147.6 ± 7.9	40.2 ± 10.7	15.0 (14.3, 15.7)*	2.020 (1.944, 2.097)*
2022	61	148.6 ± 7.4	41.4 ± 9.3	16.9 (15.9, 18.0)	2.211 (2.137, 2.285)
12–13 years					
2014	71	157.2 ± 5.4	48.9 ± 8.9	22.2 (21.1, 23.3)	2.456 (2.358, 2.554)
2015	73	157.6 ± 5.9	51.0 ± 8.9	21.9 (20.7, 23.1)	2.378 (2.263, 2.493)
2016	72	157.3 ± 5.8	47.8 ± 8.5	20.7 (19.6, 21.7)	2.308 (2.196, 2.421)
2017	75	157.1 ± 6.0	48.9 ± 9.3	20.2 (19.3, 21.2)*	2.251 (2.164, 2.337)
2018	61	155.9 ± 7.8	48.9 ± 9.9	19.5 (18.4, 20.5)*	2.202 (2.098, 2.305)*
2019	70	157.1 ± 6.6	52.2 ± 11.8	20.9 (19.7, 22.0)	2.264 (2.179, 2.350)
2022	51	158.3 ± 5.4	50.3 ± 8.8	21.9 (21.0, 22.9)	2.381 (2.275, 2.488)
14–15 years					
2014	61	160.1 ± 4.5	52.4 ± 8.8	24.1 (22.8, 25.5)	2.531 (2.385, 2.677)
2015	67	160.1 ± 5.1	55.2 ± 11.4	24.1 (23.0, 25.2)	2.482 (2.376, 2.589)
2016	88	160.4 ± 5.2	54.7 ± 11.5	22.9 (22.0, 23.8)	2.360 (2.265, 2.455)
2017	68	161.1 ± 5.0	53.3 ± 8.4	22.3 (21.2, 23.3)	2.288 (2.190, 2.386)
2018	66	162.6 ± 4.7	55.2 ± 8.6	23.3 (22.3, 24.2)	2.312 (2.232, 2.392)
2019	61	160.6 ± 4.4	53.5 ± 11.5	22.3 (21.3, 23.2)	2.305 (2.222, 2.387)
2022	46	161.8 ± 4.2	53.5 ± 9.5	23.8 (22.6, 25.1)	2.418 (2.321, 2.514)
16–17 years					
2014	71	160.8 ± 5.3	56.1 ± 9.4	26.0 (24.7, 27.3)	2.642 (2.524, 2.760)**
2015	74	161.2 ± 4.6	58.3 ± 10.1	25.9 (25.0, 26.8)	2.586 (2.484, 2.688)**
2016	75	161.6 ± 5.2	56.5 ± 10.8	23.6 (22.6, 24.5)	2.362 (2.271, 2.453)
2017	77	161.0 ± 4.7	57.5 ± 9.9	24.1 (23.1, 25.1)	2.416 (2.317, 2.514)
2018	54	160.5 ± 4.5	54.5 ± 8.2	21.8 (21.2, 22.4)*	2.247 (2.183, 2.311)
2019	66	162.2 ± 5.5	57.2 ± 11.1	22.6 (21.7, 23.5)*	2.236 (2.147, 2.325)
2022	31	164.4 ± 4.8	60.3 ± 15.5	24.5 (23.2, 25.7)	2.326 (2.183, 2.468)
18–19 years					
2014	55	161.4 ± 6.2	55.4 ± 10.5	26.4 (25.6, 27.3)**	2.664 (2.596, 2.732)**
2015	67	160.3 ± 4.8	56.6 ± 9.5	25.4 (24.3, 26.5)	2.591 (2.470, 2.713)**
2016	61	162.6 ± 5.9	57.3 ± 9.7	23.9 (22.5, 25.3)	2.362 (2.219, 2.504)
2017	54	161.2 ± 4.5	57.1 ± 10.3	23.8 (22.6, 25.0)	2.388 (2.276, 2.500)
2018	68	161.8 ± 4.9	55.8 ± 8.3	22.7 (20.9, 24.5)*	2.282 (2.132, 2.432)
2019	62	162.7 ± 5.2	59.3 ± 13.0	23.4 (22.5, 24.3)*	2.289 (2.193, 2.386)*
2022	38	164.3 ± 5.3	58.2 ± 10.3	25.0 (24.3, 25.8)	2.413 (2.350, 2.476)

Data are presented as mean ± standard deviation or marginal mean (95% confidence interval); * denotes statistically lower values compared to 2022 (reference year); ** denotes statistically higher values compared to 2022 (reference year).

Handgrip strength was higher in 2014 (1.4 kg; *p* = 0.015) for 18–19-year-olds.

### Male normalized handgrip strength

Significant omnibus effects across survey years were found for males of all age groups (*p* = 0.013 to *p* < 0.001).

Handgrip strength was lower in: (a) 2019 for 12–13-year-olds and 16–17-year-olds (−0.197 and −0.255 units; *p* = 0.029 and 0.006); and (b) 2018 for 12–13-year-olds and 16–17-year-olds (−0.259 and −0.267 units; *p* = 0.006 and 0.004).

Handgrip strength was higher in: (a) 2015 for 10–11-year-olds, 14–15-year-olds, and 18–19-year-olds (0.222 to 0.309 units; *p* = 0.012 to < 0.001); and (b) 2014 for 10–11-year-olds, 14–15-year-olds, 16–17-year-olds, and 18–19-year-olds (0.175 to 0.303 units; *p* = 0.017 to < 0.001).

### Female normalized handgrip strength

Significant omnibus effects across survey years were found for females of all age groups (*p* = 0.013 to *p* < 0.001), but none of the pairwise comparisons were significant (*p* = 0.066 to 0.376) for 14–15-year-olds.

Handgrip strength was lower in: (a) 2019 for 10–11-year-olds and 18–19-year-olds (−0.123 and −0.191 units; *p* = 0.036 and < 0.001); (b) 2018 for 10–11-year-olds and 12–13-year-olds (−0.180 and −0.302 units; *p* = 0.018 and < 0.001); and (c) 2016 for 10–11-year-olds (−0.132 units; *p* = 0.024).

Handgrip strength was higher in: (a) 2015 for 16–17-year-olds and 18–19-year-olds (0.178 and 0.260 units; *p* = 0.011 and 0.004); and (b) 2014 for 16–17-year-olds and 18–19-year-olds (0.251 and 0.316 units; *p* = 0.004 and < 0.001).

### Handgrip strength reference values

For males, reference values were the lowest for 10–11-year-olds (5th percentile = 11 kg; 50th percentile = 17 kg; 95th percentile = 24 kg; Table 3), increased for each subsequent group and were the highest for 18–19-year-olds (5th percentile = 27 kg; 50th percentile = 38 kg; 95th percentile = 51 kg).

**Table 3:** Reference values for handgrip strength for adolescent males including KNHANES waves 2014–2019 and 2022 (pooled *n* = 2,574).

Age (years)	*n*	Weighted percentile (kg)										
		5^th^	10^th^	20^th^	30^th^	40^th^	50^th^	60^th^	70^th^	80^th^	90^th^	95^th^
Handgrip strength (kg)												
10–11 years	619	11	12	14	15	16	17	17	19	20	22	24
12–13 years	558	15	17	19	21	23	25	27	29	32	35	39
14–15 years	502	23	26	28	30	32	34	36	37	40	42	46
16–17 years	458	26	29	32	34	36	37	39	41	44	47	50
18–19 years	437	27	30	33	35	37	38	40	42	45	48	51
Body size normalized handgrip strength (kg/(m^2^ * kg^0.333^)												
10–11 years	619	1.574	1.705	1.900	2.027	2.134	2.232	2.320	2.426	2.578	2.772	2.978
12–13 years	558	1.762	1.900	2.103	2.232	2.369	2.526	2.673	2.817	2.986	3.248	3.524
14–15 years	502	2.117	2.253	2.488	2.648	2.792	2.903	3.022	3.185	3.335	3.588	3.806
16–17 years	458	2.166	2.406	2.641	2.788	2.927	3.054	3.220	3.391	3.595	3.777	4.008
18–19 years	437	2.308	2.478	2.704	2.889	3.008	3.097	3.204	3.364	3.559	3.820	4.007

For females, reference values were the lowest for 10–11-year-olds (5th percentile = 10 kg; 50th percentile = 15 kg; 95th percentile = 23 kg; Table 4), increased for each subsequent group and were the highest for 16–17-year-olds and 18–19-year-olds (5th percentile = 17 kg; 50th percentile = 25 kg; 95th percentile = 32 kg).

**Table 4:** Reference values for handgrip strength for adolescent females including KNHANES waves 2014–2019 and 2022 (pooled *n* = 2,293).

Age (years)	*n*	Weighted percentile (kg)										
		5^th^	10^th^	20^th^	30^th^	40^th^	50^th^	60^th^	70^th^	80^th^	90^th^	95^th^
Handgrip strength (kg)												
10–11 years	510	10	11	12	13	14	15	16	17	19	21	23
12–13 years	473	14	16	17	19	19	21	22	23	25	27	29
14–15 years	457	17	18	19	21	22	23	24	25	27	29	31
16–17 years	448	17	19	20	21	23	24	25	26	28	30	32
18–19 years	405	16	19	21	22	23	25	26	27	28	30	32
Body size normalized handgrip strength (kg/(m^2^ * kg^0.333^)												
10–11 years	510	1.470	1.601	1.773	1.881	1.995	2.102	2.189	2.317	2.455	2.680	2.804
12–13 years	473	1.701	1.828	1.997	2.090	2.211	2.300	2.402	2.541	2.684	2.867	3.020
14–15 years	457	1.685	1.872	2.050	2.158	2.260	2.386	2.509	2.611	2.732	2.924	3.037
16–17 years	448	1.782	1.895	2.058	2.178	2.286	2.376	2.510	2.631	2.819	3.032	3.157
18–19 years	405	1.595	1.881	2.115	2.250	2.339	2.463	2.552	2.658	2.775	2.974	3.130

### Normalized handgrip strength reference values

For males, reference values were the lowest for 10–11-year-olds (5th percentile = 1.574 units; 50th percentile = 2.232 units; 95th percentile = 2.978 units), increased for each subsequent group and were the highest for 18–19-year-olds (5th percentile = 2.308 units; 50th percentile = 3.097 units; 95th percentile = 4.007 units).

For females, reference values were the lowest for 10–11-year-olds (5th percentile = 1.470 units; 50th percentile = 2.102 units; 95th percentile = 2.804 units), increased for each subsequent group and were the highest for 16–17-year-olds and 18–19-year-olds (5th percentile = 1.782 units; 50th percentile = 2.463 units; 95th percentile = 3.157 units).

### Secondary analyses

Secondary analyses including all six handgrip strength attempts for the 2014–2019 waves are provided in the following supplementary files:

- Supplementary File 1 in S1 Data: Temporal changes in handgrip strength among males
- Supplementary File 2 in S1 Data: Temporal changes in handgrip strength among females
- Supplementary File 3 in S1 Data: Reference values for males
- Supplementary File 4 in S1 Data: Reference values for females

Separate analyses based on the most recent wave (2022) are presented in:

- Supplementary File 5 in S1 Data: Reference values for males
- Supplementary File 6 in S1 Data: Reference values for females

## Discussion

We found that handgrip strength remained fairly stable over the 9-year period analyzed, even though in some of the years it was lower or higher compared to the most recently available wave. However, these changes were generally small, did not occur at every survey year, and were completely absent for some of the sex and age groups. When exploring normalized handgrip strength, a generally similar pattern occurred even though the differences between specific years were not necessarily uniform with absolute handgrip strength.

While handgrip strength increased from 1960 to 2017 [13], some of the more recent studies reported a decline. For example, in a sample of 1,004 Brazilian adolescents, there was a 2.5–3 kg decline in median handgrip strength between 2007 and 2017–2018 [27]. In Macao, a decline in handgrip strength by ~3 kg was also observed among 1,980 adolescents who were evaluated in 2005, 2010, 2015, and 2020 [28]. At least for some of the age groups, our results are similar, as we observed a decline compared to 2022. Interestingly, this decline most consistently occurred in 2019 and 2018, which are years preceding the COVID-19 pandemic. Other studies reported that aspects of physical fitness declined after the pandemic [29,30], but this does not seem to be the case for handgrip strength of adolescents in the Republic of Korea. However, as only one datapoint was available post-COVID-19, future research will need to periodically update the evidence in the following years to establish further changes as the absence of 2020–2021 in our data restricts pandemic-specific conclusions. Studies exploring temporal changes generally focused only on absolute but not normalized handgrip strength [27,28]. Here, we normalized handgrip strength for body size, using methods specifically derived for adolescent populations. A similar pattern of differences was generally found, even though it did not completely mirror the results for absolute handgrip strength. These findings highlight that future studies may consider using normalized handgrip strength if height and weight data are available, to account for possible differences in body size across different survey/cohort waves.

In Colombian adolescents, reference values were generally lower compared to our data [31]. For example, the 50th percentiles for late adolescent males were 29–33 kg, whereas in our sample, the 50th percentiles were 34–38 kg. Differences were observed for other percentiles as well; we found higher handgrip strength at the lowest and highest percentiles, for both sexes. Reference values were generally higher among Spanish adolescents compared to ours, with some of the percentile values even exceeding 5 kg [32]. However, there are inherent limitations in these between-studies comparisons due to their methodological differences. For instance, methodological differences included the use of verbal encouragement and whether testing was conducted on both hands or restricted to the right hand. Additionally, the Spanish study did not provide normalized reference values, while the Colombian study normalized only for body mass, limiting any further comparison to our results. As normalization for height and body mass appears to be most appropriate for the adolescent population [26], future studies should consider presenting both absolute and body size normalized handgrip strength to facilitate cross-country comparisons.

Nevill et al. [26], provided a method for normalizing handgrip strength according to body size and are also the only ones that provided reference values for this metric. In their study, data from National Health and Nutrition Examination Survey was used, and reference values were calculated for youth aged 6–18 years (*n* = 4,816). The normalized handgrip strength reference values for this sample were generally higher compared to the ones generated herein, both for males and females. Again, while there are methodological differences between the studies (e.g., start with the dominant hand vs. random start with either hand), these data suggest that Western populations may have higher handgrip strength compared to Asian populations, even when normalized for height and body mass. These comparisons underscore the importance of using population-specific reference values for handgrip strength. Additionally, the protocols used to obtain handgrip strength should be standardized in line with the current recommendations [25], as this would enable a more straightforward comparison of findings between studies.

The KNHANES dataset was previously used to explore temporal changes in handgrip strength and provide reference values. In the only study on temporal changes, the authors found a decline in handgrip strength when analyzing data from 2014–2017 [33]. However, it is worth noting that this analysis covered only a four-year period. Our analysis extends these findings as it spanned 9 years and included a data point from the post-COVID-19 period (2022 wave). Two studies also previously used the KNHANES to establish reference values for handgrip strength [23,24]. However, both analyses only provided average (mean) values, not percentiles. Averages have a limited utility, as they do not provide identification of a specific position across the population, which is accomplished with percentile values. Additionally, the two previous analyses on reference values used only KNHANES waves 2014–2017.

### Secondary analyses

In one of the secondary analyses, we examined temporal changes and calculated reference values while considering all six attempts across waves 2014–2019. The results for temporal changes largely remained the same. For example, for male handgrip strength, all of the same pairwise comparisons remained significant. For normalized handgrip strength, one comparison was no longer significant (i.e., the 2019 decline among 16–17-year-olds), while a significant decline was observed in 2016 among 10–11-year-olds. While some findings slightly differed, they did not overall change the main interpretation of the primary findings. With the inclusion of the third attempt, the reference values remained the same (34/55 percentiles for females and 27/55 percentiles for males) or were higher by 1 kg.

As a part of the secondary analysis, we also calculated reference values using only the 2022 wave. This approach was adopted because there were temporal changes in some of the survey years and therefore, pooling all waves into a single analysis to calculate reference values may introduce variability. When comparing the most recent wave with the pooled analysis, we found that 62–78% of the reference values were within ± 1 kg. Larger differences observed in some values likely reflect the smaller sample size of the single wave (*n* = 481). Ultimately, there will always be a trade-off between the approaches – pooling of multiple waves will produce higher sample sizes and likely greater precision, while the use of the most recent data is likely to more accurately reflect the current population.

### Strengths, limitations, and considerations

The strengths of this study include the overall large sample size and nationally representative data. Despite these strengths, several limitations need to be mentioned. Specifically, while the handgrip strength protocol was generally standardized across the waves, the number of attempts was reduced from six to four in the 2022 wave. We accounted for this issue by using only the first four attempts from waves 2014–2019 and by conducting additional analyses with all six attempts, but this still needs to be highlighted as a limitation. Additionally, handgrip strength was not collected as a part of the KNHANES 2020–2021 waves. Thus, the potential influence of these two years on the temporal changes remains unclear. While this study explored changes over the survey years, we did not examine the influence of variables such as physical activity, diet, or other behavioural factors on handgrip strength. These aspects should be further explored in future research. Even though body mass and height were used to normalize handgrip strength, muscle mass was not considered. While not a limitation per se, it is worth noting that confidence intervals for percentiles were not calculated because the primary purpose of percentiles is to describe the empirical distribution rather than to estimate parameters for inference. However, future studies may consider calculating confidence intervals to show sampling variability. Finally, another consideration is that the reference values represent population-based normative data but not clinically validated cut-offs. Further studies associating the reference values to clinical outcomes are needed to establish their diagnostic utility.

## Conclusion

Handgrip strength remained relatively stable over the 9-year period, even though in some of the years it was lower or higher compared to the most recently available wave. However, these differences were inconsistent across survey years and absent in some sex–age groups. A similar pattern was observed for normalized handgrip strength, although the specific differences did not always align with the absolute values. The provided reference values enable interpretation of handgrip strength in Korean adolescents.

## Supporting information

S1 Data Secondary analyses. (ZIP)

## Abbreviations

CSGLM: Complex Samples General Linear Model
KNHANES: Korea National Health and Nutrition Examination Survey
PSU: primary sampling units
