# Supplementary material for: Temporal changes and reference values for adolescent handgrip strength in the Republic of Korea: A 9-year analysis of the KNHANES cohort
Source: PLoS One. 2026 Sep 3;21(9):e0357474. doi: 10.1371/journal.pone.0357474 (PMC13541116; doi:10.1371/journal.pone.0357474)
Supplement: Supplementary file 1 [file pone.0357474.s001.zip › Supplementary file 3.docx]

**Supplementary file 3.** Reference values for handgrip strength for adolescent males including KNHANES waves 2014–2019 and 2022 (pooled *n* = 2,574), while considering all six attempts in the 2014–2019 waves

| **Age (years)** | ***n*** | **Weighted percentile** **(kg)** | | | | | | | | | | |
| --- | --- | --- | --- | --- | --- | --- | --- | --- | --- | --- | --- | --- |
|  |  | **5^th^** | **10^th^** | **20^th^** | **30^th^** | **40^th^** | **50^th^** | **60^th^** | **70^th^** | **80^th^** | **90^th^** | **95^th^** |
| Handgrip strength (kg) | | | | | | | | | | | | |
| 10–11 years | 619 | 12 | 13 | 14 | 15 | 16 | 17 | 18 | 19 | 20 | 22 | 25 |
| 12–13 years | 558 | 16 | 17 | 20 | 21 | 23 | 25 | 28 | 29 | 32 | 35 | 39 |
| 14–15 years | 502 | 24 | 26 | 29 | 31 | 33 | 34 | 36 | 38 | 40 | 43 | 46 |
| 16–17 years | 458 | 26 | 30 | 33 | 35 | 36 | 37 | 39 | 42 | 44 | 48 | 51 |
| 18–19 years | 437 | 28 | 31 | 34 | 35 | 37 | 39 | 41 | 43 | 45 | 49 | 52 |
| Body size normalized handgrip strength (kg/(m^2^ ∗ kg^0.333^) | | | | | | | | | | | | |
| 10–11 years | 619 | 1.632 | 1.739 | 1.951 | 2.072 | 2.175 | 2.259 | 2.361 | 2.459 | 2.608 | 2.794 | 2.990 |
| 12–13 years | 558 | 1.793 | 1.935 | 2.132 | 2.261 | 2.386 | 2.569 | 2.703 | 2.859 | 3.015 | 3.289 | 3.545 |
| 14–15 years | 502 | 2.140 | 2.300 | 2.529 | 2.691 | 2.826 | 2.919 | 3.073 | 3.205 | 3.403 | 3.613 | 3.806 |
| 16–17 years | 458 | 2.217 | 2.448 | 2.667 | 2.822 | 2.968 | 3.066 | 3.241 | 3.434 | 3.617 | 3.801 | 4.057 |
| 18–19 years | 437 | 2.308 | 2.484 | 2.755 | 2.926 | 3.039 | 3.121 | 3.248 | 3.447 | 3.598 | 3.846 | 4.038 |
| 18–19 years | 437 | 2.308 | 2.478 | 2.704 | 2.889 | 3.008 | 3.097 | 3.204 | 3.364 | 3.559 | 3.820 | 4.007 |
