# Supplementary material for: Temporal changes and reference values for adolescent handgrip strength in the Republic of Korea: A 9-year analysis of the KNHANES cohort
Source: PLoS One. 2026 Sep 3;21(9):e0357474. doi: 10.1371/journal.pone.0357474 (PMC13541116; doi:10.1371/journal.pone.0357474)
Supplement: Supplementary file 1 [file pone.0357474.s001.zip › Supplementary file 4.docx]

**Supplementary file 4.** Reference values for handgrip strength for adolescent females including KNHANES waves 2014–2019 and 2022 (pooled *n* = 2,293), while considering all six attempts in the 2014–2019 waves

| **Age (years)** | ***n*** | **Weighted percentile** **(kg)** | | | | | | | | | | |
| --- | --- | --- | --- | --- | --- | --- | --- | --- | --- | --- | --- | --- |
|  |  | **5^th^** | **10^th^** | **20^th^** | **30^th^** | **40^th^** | **50^th^** | **60^th^** | **70^th^** | **80^th^** | **90^th^** | **95^th^** |
| Handgrip strength (kg) | | | | | | | | | | | | |
| 10–11 years | 510 | 10 | 11 | 12 | 13 | 14 | 15 | 16 | 18 | 20 | 22 | 23 |
| 12–13 years | 473 | 15 | 16 | 18 | 19 | 20 | 21 | 22 | 24 | 25 | 27 | 29 |
| 14–15 years | 457 | 17 | 18 | 20 | 21 | 22 | 23 | 25 | 26 | 27 | 29 | 32 |
| 16–17 years | 448 | 17 | 19 | 21 | 22 | 23 | 24 | 26 | 27 | 28 | 30 | 33 |
| 18–19 years | 405 | 17 | 19 | 21 | 23 | 24 | 25 | 26 | 28 | 28 | 31 | 32 |
| Body size normalized handgrip strength (kg/(m^2^ ∗ kg^0.333^) | | | | | | | | | | | | |
| 10–11 years | 510 | 1.482 | 1.620 | 1.791 | 1.931 | 2.021 | 2.117 | 2.221 | 2.330 | 2.481 | 2.683 | 2.845 |
| 12–13 years | 473 | 1.730 | 1.873 | 2.011 | 2.135 | 2.239 | 2.337 | 2.424 | 2.552 | 2.692 | 2.870 | 3.049 |
| 14–15 years | 457 | 1.747 | 1.909 | 2.084 | 2.185 | 2.294 | 2.395 | 2.521 | 2.631 | 2.760 | 2.947 | 3.056 |
| 16–17 years | 448 | 1.790 | 1.920 | 2.089 | 2.194 | 2.294 | 2.410 | 2.526 | 2.645 | 2.835 | 3.054 | 3.179 |
| 18–19 years | 405 | 1.693 | 1.911 | 2.133 | 2.264 | 2.350 | 2.493 | 2.567 | 2.695 | 2.811 | 3.038 | 3.212 |
