# Supplementary material for: Temporal changes and reference values for adolescent handgrip strength in the Republic of Korea: A 9-year analysis of the KNHANES cohort
Source: PLoS One. 2026 Sep 3;21(9):e0357474. doi: 10.1371/journal.pone.0357474 (PMC13541116; doi:10.1371/journal.pone.0357474)
Supplement: Supplementary file 1 [file pone.0357474.s001.zip › Supplementary file 5.docx]

**Supplementary file 5.** Reference values for handgrip strength for adolescent males including only the 2022 KNHANES wave (pooled *n* = 254)

| **Age (years)** | ***n*** | **Weighted percentile** **(kg)** | | | | | | | | | | |
| --- | --- | --- | --- | --- | --- | --- | --- | --- | --- | --- | --- | --- |
|  |  | **5^th^** | **10^th^** | **20^th^** | **30^th^** | **40^th^** | **50^th^** | **60^th^** | **70^th^** | **80^th^** | **90^th^** | **95^th^** |
| Handgrip strength (kg) | | | | | | | | | | | | |
| 10–11 years | 68 | 13 | 14 | 14 | 16 | 16 | 17 | 17 | 18 | 20 | 23 | 25 |
| 12–13 years | 54 | 17 | 17 | 21 | 24 | 26 | 28 | 30 | 30 | 32 | 35 | 36 |
| 14–15 years | 53 | 23 | 24 | 27 | 29 | 31 | 32 | 34 | 36 | 39 | 42 | 45 |
| 16–17 years | 41 | 29 | 31 | 33 | 35 | 37 | 39 | 41 | 42 | 45 | 49 | 53 |
| 18–19 years | 38 | 29 | 31 | 33 | 35 | 36 | 37 | 40 | 43 | 46 | 49 | 49 |
| Body size normalized handgrip strength (kg/(m^2^ ∗ kg^0.333^) | | | | | | | | | | | | |
| 10–11 years | 68 | 1.633 | 1.699 | 1.872 | 1.959 | 2.049 | 2.153 | 2.218 | 2.334 | 2.525 | 2.682 | 2.770 |
| 12–13 years | 54 | 1.794 | 1.984 | 2.168 | 2.232 | 2.317 | 2.608 | 2.642 | 2.981 | 3.246 | 3.524 | 3.659 |
| 14–15 years | 53 | 2.162 | 2.190 | 2.518 | 2.556 | 2.693 | 2.904 | 2.937 | 3.094 | 3.257 | 3.418 | 3.480 |
| 16–17 years | 41 | 2.267 | 2.404 | 2.679 | 2.853 | 2.964 | 3.066 | 3.250 | 3.476 | 3.613 | 3.680 | 4.008 |
| 18–19 years | 38 | 2.415 | 2.597 | 2.755 | 2.856 | 2.917 | 2.943 | 3.077 | 3.188 | 3.378 | 3.604 | 3.756 |
