# Supplementary material for: Temporal changes and reference values for adolescent handgrip strength in the Republic of Korea: A 9-year analysis of the KNHANES cohort
Source: PLoS One. 2026 Sep 3;21(9):e0357474. doi: 10.1371/journal.pone.0357474 (PMC13541116; doi:10.1371/journal.pone.0357474)
Supplement: Supplementary file 1 [file pone.0357474.s001.zip › Supplementary file 6.docx]

**Supplementary file 6.** Reference values for handgrip strength for adolescent females including only the 2022 KNHANES wave (pooled *n* = 227)

| **Age (years)** | ***n*** | **Weighted percentile** **(kg)** | | | | | | | | | | |
| --- | --- | --- | --- | --- | --- | --- | --- | --- | --- | --- | --- | --- |
|  |  | **5^th^** | **10^th^** | **20^th^** | **30^th^** | **40^th^** | **50^th^** | **60^th^** | **70^th^** | **80^th^** | **90^th^** | **95^th^** |
| Handgrip strength (kg) | | | | | | | | | | | | |
| 10–11 years | 61 | 11 | 12 | 13 | 14 | 15 | 16 | 17 | 19 | 21 | 23 | 24 |
| 12–13 years | 51 | 15 | 16 | 18 | 19 | 21 | 22 | 23 | 24 | 26 | 27 | 29 |
| 14–15 years | 46 | 15 | 18 | 20 | 21 | 23 | 23 | 25 | 25 | 26 | 30 | 35 |
| 16–17 years | 31 | 17 | 18 | 20 | 21 | 22 | 24 | 25 | 26 | 28 | 33 | 35 |
| 18–19 years | 38 | 21 | 22 | 22 | 23 | 24 | 25 | 25 | 27 | 28 | 28 | 31 |
| Body size normalized handgrip strength (kg/(m^2^ ∗ kg^0.333^) | | | | | | | | | | | | |
| 10–11 years | 61 | 1.554 | 1.639 | 1.859 | 1.990 | 2.043 | 2.187 | 2.282 | 2.429 | 2.567 | 2.792 | 2.941 |
| 12–13 years | 51 | 1.638 | 1.787 | 1.924 | 2.170 | 2.246 | 2.347 | 2.469 | 2.625 | 2.825 | 2.940 | 3.229 |
| 14–15 years | 46 | 1.752 | 1.893 | 2.042 | 2.154 | 2.346 | 2.476 | 2.580 | 2.639 | 2.759 | 2.894 | 3.120 |
| 16–17 years | 31 | 1.850 | 1.863 | 1.949 | 1.981 | 2.089 | 2.196 | 2.291 | 2.543 | 2.858 | 3.032 | 3.121 |
| 18–19 years | 38 | 1.729 | 2.073 | 2.144 | 2.269 | 2.339 | 2.423 | 2.520 | 2.551 | 2.609 | 2.795 | 2.842 |
