# Supplementary material for: Temporal changes and reference values for adolescent handgrip strength in the Republic of Korea: A 9-year analysis of the KNHANES cohort
Source: PLoS One. 2026 Sep 3;21(9):e0357474. doi: 10.1371/journal.pone.0357474 (PMC13541116; doi:10.1371/journal.pone.0357474)
Supplement: Supplementary file 1 [file pone.0357474.s001.zip › Supplementary file 1.docx]

**Supplementary file 1.** Male participant handgrip strength values according to survey year (*n* = 2,574) while considering all six attempts in the 2014–2019 waves

| **Year** | ***n*** | **Handgrip strength (kg)** | **Normalized handgrip strength (kg/(m^2^ ∗ kg^0.333^)** |
| --- | --- | --- | --- |
| 10–11 years | | | |
| 2014 | 88 | 17.8 (17.0, 18.6) | 2.427 (2.332, 2.522)** |
| 2015 | 91 | 18.7 (17.9, 19.5) | 2.438 (2.361, 2.515)** |
| 2016 | 89 | 17.2 (16.3, 18.2) | 2.332 (2.269, 2.396)** |
| 2017 | 85 | 17.5 (16.7, 18.3) | 2.238 (2.152, 2.325) |
| 2018 | 98 | 16.1 (15.2, 16.9)* | 2.140 (2.045, 2.235) |
| 2019 | 100 | 15.9 (15.2, 16.6)* | 2.133 (2.054, 2.211) |
| 2022 | 68 | 17.6 (16.9, 18.4) | 2.183 (2.101, 2.265) |
| 12–13 years | | | |
| 2014 | 71 | 27.6 (26.1, 29.2) | 2.782 (2.653, 2.911) |
| 2015 | 87 | 26.4(24.8, 28.0) | 2.665 (2.561, 2.769) |
| 2016 | 91 | 26.3 (24.8, 27.7) | 2.609 (2.475, 2.743) |
| 2017 | 93 | 25.4 (24.0, 26.8) | 2.563 (2.467, 2.659) |
| 2018 | 82 | 24.2 (22.2, 26.1)* | 2.405 (2.292, 2.519)* |
| 2019 | 80 | 24.5 (23.0, 26.0)* | 2.421 (2.301, 2.541)* |
| 2022 | 54 | 27.0 (25.5, 28.4) | 2.634 (2.492, 2.776) |
| 14–15 years | | | |
| 2014 | 89 | 36.8 (35.3, 38.3)** | 3.162 (3.050, 3.274)** |
| 2015 | 86 | 35.9 (34.4, 37.5)** | 3.181 (3.077, 3.285)** |
| 2016 | 76 | 33.6 (32.0, 35.1) | 2.864 (2.718, 3.009) |
| 2017 | 63 | 33.8 (32.0, 35.6) | 2.862 (2.750, 2.974) |
| 2018 | 64 | 32.5 (31.3, 33.7) | 2.733 (2.620, 2.846) |
| 2019 | 71 | 33.8 (32.4, 35.2) | 2.882 (2.782, 2.982) |
| 2022 | 53 | 32.9 (31.2, 34.5) | 2.828 (2.706, 2.950) |
| 16–17 years | | | |
| 2014 | 68 | 40.3 (38.7, 41.8) | 3.364 (3.269, 3.460)** |
| 2015 | 66 | 40.6 (38.7, 42.6) | 3.308 (3.142, 3.475) |
| 2016 | 74 | 35.7 (34.2, 37.3)* | 3.009 (2.869, 3.148) |
| 2017 | 80 | 38.4 (36.9, 40.0) | 3.171 (3.047, 3.296) |
| 2018 | 68 | 35.8 (33.9, 37.6)* | 2.902 (2.787, 3.017)* |
| 2019 | 61 | 37.4 (36.0, 38.9) | 2.978 (2.864, 3.092) |
| 2022 | 41 | 39.4 (36.5, 42.3) | 3.127 (2.993, 3.262) |
| 18–19 years | | | |
| 2014 | 62 | 39.5 (38.5, 40.6) | 3.250 (3.161, 3.339)** |
| 2015 | 83 | 40.9 (39.1, 42.6) | 3.322 (3.182, 3.462)** |
| 2016 | 65 | 38.7 (37.2, 40.3) | 3.126 (3.017, 3.235) |
| 2017 | 60 | 40.0 (38.2, 41.8) | 3.191 (3.095, 3.288) |
| 2018 | 62 | 39.0 (37.4, 40.6) | 3.078 (2.955, 3.201) |
| 2019 | 67 | 38.9 (36.8, 41.0) | 3.053 (2.903, 3.204) |
| 2022 | 38 | 38.9 (36.6, 41.1) | 3.048 (2.938, 3.159) |
| Data are presented as mean ± marginal mean (95% confidence interval); * denotes statistically lower values compared to 2022 (reference year); ** denotes statistically higher values compared to 2022 (reference year) | | | |
