# Supplementary material for: Temporal changes and reference values for adolescent handgrip strength in the Republic of Korea: A 9-year analysis of the KNHANES cohort
Source: PLoS One. 2026 Sep 3;21(9):e0357474. doi: 10.1371/journal.pone.0357474 (PMC13541116; doi:10.1371/journal.pone.0357474)
Supplement: Supplementary file 1 [file pone.0357474.s001.zip › Supplementary file 2.docx]

**Supplementary file 2.** Female participant handgrip strength values according to survey year (*n* = 2,293) while considering all six attempts in the 2014-2019 waves

| **Year** | ***n*** | **Handgrip strength (kg)** | **Normalized handgrip strength (kg/(m^2^ ∗ kg^0.333^)** |
| --- | --- | --- | --- |
| 10–11 years | | | |
| 2014 | 69 | 16.6 (15.4, 17.7) | 2.236 (2.138, 2.334) |
| 2015 | 60 | 17.4 (16.1, 18.6) | 2.318 (2.198, 2.439) |
| 2016 | 78 | 15.6 (14.8, 16.5) | 2.130 (2.037, 2.223) |
| 2017 | 91 | 15.7 (15.0, 16.4)* | 2.125 (2.050, 2.201) |
| 2018 | 75 | 14.9 (13.8, 16.0)* | 1.944 (1.834, 2.053)* |
| 2019 | 76 | 15.2 (14.5, 15.8)* | 2.045 (1.971, 2.118)* |
| 2022 | 61 | 16.9 (15.9, 18.0) | 2.211 (2.137, 2.285) |
| 12–13 years | | | |
| 2014 | 71 | 22.3 (21.2, 23.4) | 2.469 (2.372, 2.567) |
| 2015 | 73 | 22.2 (21.0, 23.5) | 2.409 (2.293, 2.525) |
| 2016 | 72 | 21.0 (19.9, 22.0) | 2.342 (2.234, 2.449) |
| 2017 | 75 | 20.6 (19.6, 21.5) | 2.288 (2.200, 2.375) |
| 2018 | 61 | 19.7 (18.6, 20.7)* | 2.224 (2.120, 2.328)* |
| 2019 | 70 | 21.1 (20.0, 22.2) | 2.292 (2.206, 2.378) |
| 2022 | 51 | 21.9 (21.0, 22.9) | 2.381 (2.275, 2.488) |
| 14–15 years | | | |
| 2014 | 61 | 24.3 (22.9, 25.7) | 2.547 (2.399, 2.695) |
| 2015 | 67 | 24.5 (23.5, 25.5) | 2.522 (2.419, 2.625) |
| 2016 | 88 | 23.1 (22.3, 24.0) | 2.388 (2.294, 2.482) |
| 2017 | 68 | 22.8 (21.7, 23.8) | 2.338 (2.242, 2.434) |
| 2018 | 66 | 23.5 (22.6, 24.5) | 2.335 (2.258, 2.413) |
| 2019 | 61 | 22.8 (21.7, 23.9) | 2.359 (2.256, 2.462) |
| 2022 | 46 | 23.8 (22.6, 25.1) | 2.418 (2.321, 2.514) |
| 16–17 years | | | |
| 2014 | 71 | 26.1 (24.9, 27.4) | 2.659 (2.547, 2.770)** |
| 2015 | 74 | 26.2 (25.2, 27.1)** | 2.613 (2.507, 2.719)** |
| 2016 | 75 | 23.9 (23.0, 24.8) | 2.394 (2.309, 2.479) |
| 2017 | 77 | 24.3 (23.4, 25.3) | 2.442 (2.344, 2.540) |
| 2018 | 54 | 22.1 (21.5, 22.7)* | 2.274 (2.212, 2.335) |
| 2019 | 66 | 22.7 (21.8, 23.6)* | 2.250 (2.162, 2.338) |
| 2022 | 31 | 24.5 (23.2, 25.7) | 2.326 (2.183, 2.468) |
| 18–19 years | | | |
| 2014 | 55 | 26.8 (25.9, 27.7)** | 2.697 (2.620, 2.775)** |
| 2015 | 67 | 25.7 (24.6, 26.8) | 2.620 (2.500, 2.739)** |
| 2016 | 61 | 24.4 (23.2, 25.5) | 2.412 (2.291, 2.533) |
| 2017 | 54 | 24.2 (23.0, 25.3) | 2.423 (2.318, 2.528) |
| 2018 | 68 | 23.1 (21.3, 24.9) | 2.325 (2.175, 2.474) |
| 2019 | 62 | 23.7 (22.8, 24.5)* | 2.318 (2.222, 2.413) |
| 2022 | 38 | 25.0 (24.3, 25.8) | 2.413 (2.350, 2.476) |
| Data are presented as mean ± marginal mean (95% confidence interval); * denotes statistically lower values compared to 2022 (reference year); ** denotes statistically higher values compared to 2022 (reference year) | | | |
